# Influence of Modification on Flow Stress Behavior and Corrosive Properties of a Hypoeutectic Al-Si Alloy

**DOI:** 10.3390/ma15072697

**Published:** 2022-04-06

**Authors:** Kai Du, Liying Song, Shaohui Huang, Xiaoguang Yuan

**Affiliations:** 1 School of Materials Science and Engineering, Shenyang University of Technology, Shenyang 110870, China; kai.du@bmw-brilliance.cn; 2 Department of Technical Planning Press Shop, BMW Brilliance Automotive Ltd., Shenyang 100044, China; shaohui.huang@bmw-brilliance.cn; 3 The State Key Laboratory of Rolling Automation, Northeastern University, Shenyang 110819, China

**Keywords:** Al-7Si alloy, stress-strain curves, thermal compression test, corrosive properties, microstructures

## Abstract

The flow stress behavior and corrosive properties of Al-7Si alloy were studied by thermal compression test, electrochemical test, and electron probe microanalysis. The influences of temperatures, strain rates, strains, and morphology of Si particles on stress-strain curves of Al-7Si alloy were analyzed. The peak stress of unmodified-Al-7Si alloy is higher than that of Sr-modified-Al-7Si alloy at the same deformation conditions, and the phenomenon is obviously relevant to the distribution and morphology of Si particles. The morphology of Si particles of unmodified-undeformed-Al-7Si alloy is a typical thick layer, and that of unmodified-deformed-Al-7Si alloy is broken into relatively small particles and the distribution is relatively even in homogeneous deformation zone II. The distribution of Si particles in Sr-modified-deformed-Al-7Si alloy is obviously more even than undeformed alloy. The effect of a small deformation of 20% on the distribution and morphology of Si particles is obviously smaller than that of a large deformation of 50%. The electrochemical self-corrosion potential of Sr-modified-Al-7Si alloy is higher than that of unmodified-Al-7Si alloy, and it proves that the distribution and morphology of Si particles have a certain influence on the corrosive properties of Al-7Si alloy. That is, the even fine Si particles are more conducive to improving the corrosive properties of Al-7Si alloy.

## 1. Introduction

Al-Si alloys have been extensively applied to many industrial areas owing to its good fluidity, small line contraction tendency, and high corrosive properties [1,2]. Si particles play a critical role in the formability of Al-Si alloys. The size, morphology, distribution, and the number of eutectic Si particles are acknowledged to be key factors which affecting the properties of Al-Si alloys [3,4,5,6]. The presence of coarse Si particles may result in premature crack initiation in the process of deformation. Therefore, controlling the morphology and distribution of Si particles is an important way to further develop Al-Si alloys.

Several routes, such as chemical modification, solution heat treatment, and deformation, have been used to attain a fine ideal microstructure and to modify the morphology of the Si particles [7,8,9,10,11,12,13,14,15,16,17,18,19]. The chemical modification and heat treatment methods are constrained by economy, resources, and environment. In recent years, high-pressure torsion, hot rolling, equal channel angular pressing and semi-solid die casting, have been employed to obtain the ideal microstructures of Al-Si alloys [18,19,20,21,22,23,24,25]. Huang et al. [26] found that hot deformation could not only effectively eliminate casting defects, but also make the distribution of eutectic Si particles of Al-Si alloy even. Cheng et al. [1] reported that the rolled Al-Si alloy exhibited the higher strength and ductility than those of undeformed Al-Si alloy. That is mainly due to rolling refined the α(Al) grains and introducing dislocations into the alloys. Thus, deformation seems to be an effective way to improve the mechanical properties of the Al-Si alloys by changing the morphology of the Si particles. Thermoplastic deformation has become a new way of strengthening and toughening the Al-Si alloy, and the process developed on the basis of thermoplastic deformation has broad application prospects. Therefore, it is necessary to explore the effect of morphology of Si particles on the flow stress behavior and the flow stress behavior of Al-Si alloy at different conditions. The compression test is regarded as an effective method to study the deformation behavior of Al-Si alloys [27].

Wei et al. [28] reported that the intermetallic phases are cathodic with relative to the α(Al) matrix. Zeng et al. [29] found that the Si particle was always acting as the cathode causing the α(Al) matrix acting as the anode to dissolve and thus cause corrosion around it. Andreatta et al. [30] reported that the heat treatment increased the potential difference between the intermetallic and the adjacent matrix. In addition, Rafieazad et al. [31,32,33] reported that the as-cast Al-Si alloy with a coarse dendritic microstructure containing the acicular Si particles, where intermetallic constituents and second phase particles can result in mediocre corrosive properties and mechanical properties of the alloy. However, the influence of Si particles’ morphology on the corrosive properties of Al-Si alloy is rarely studied.

All in all, though many researches have been carried out to report Al-Si alloys, the effect of thermal deformation parameters on the microstructure of Al-Si alloys have not been elucidated clearly. Moreover, there is a lack of sufficient information about the influence of microstructure morphology on corrosive properties. Therefore, it is essential to study the corrosive properties and microstructures evolution of Al-7Si alloy. The research results have certain guiding significance for practical production. In this work, the influences of temperatures, strain rates, strains, and initial microstructures on stress-strain curves of Al-7Si alloy were discussed in detail. The influence of the distribution and morphology of Si particles on the corrosive properties of Al-Si alloy was further researched.

## 2. Experimental Procedures

Table 1 shows the chemical compositions of the tested alloys. The tested alloys were obtained by metal mold gravity casting, and the Al-7Si alloys were melted at a casting temperature of 750 °C. The Sr-modified-Al-7Si alloy was modified by adding 0.3%(wt.) Sr(Strontium). The raw materials are the pure Al(aluminum), pure Mg(magnesium) and Al-30Si master alloy ingots, and Sr element was added in the form of master alloy Al-10% Sr(wt.). After casting, the samples of Sr-modified-Al-7Si alloy were heat-treated to make Si phase shape spheroidization and distribution more even at 525 °C × 8 h.

The samples were etched with HF corrosive reagent to observe the stereoscopic morphology of Si-eutectic (10 mL HF + 90 mL distilled H_2_O, 10 min). In addition, the sample was etched with HF corrosive reagent to observe the microstructures of Si-eutectic at different zones at 400 °C × 0.5 s^−1^ in Al-7Si-Sr-modified alloy (0.5 mL HF + 99.5 mL distilled H_2_O, 8 s). A JXA-8530F EPMA (Electron Probe Microanalysis, JEOL, Tokyo, Japan) was applied to study the microstructures and distribution of Al and Si elements. The central homogeneous deformation zone II of the deformed samples was used to research the effects of thermal deformation on the alteration of Si particles.

**Figure 1:**
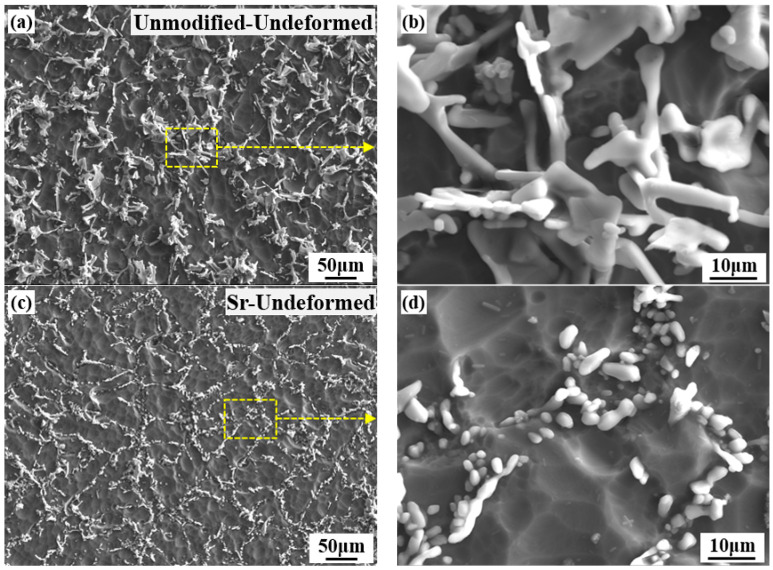
Initial microstructures of the undeformed samples: (**a**,**b**) unmodified-undeformed; (**c**,**d**) Sr-undeformed.

Researchers [34,35,36] point out that there are Si embryos in the liquid. Without the addition of Sr, the Si crystal embryo will develop into eutectic crystal nucleus, which grows in the inherent step mode and is ahead of the eutectic aluminum phase to form lamellar. When there is metamorphic element Sr in the liquid phase, the free Sr will be adsorbed on the surface of the Si embryo. When the concentration of free Sr on the surface of Si embryo reaches a certain level, the inherent step growth of Si is prevented, and the embryo cannot develop and grow. The adsorption of Sr reduces the mobility of Si embryos in the liquid phase, and the growth rate of Si particles is lower than that of the α(Al) phase. The α(Al) phase grows in advance, and the Si particles grows in the “nodes” between the α(Al) crystals by twinning, forming the fibrous eutectic structure. The initial microstructures which consist of α(Al) and eutectic Si particles of the undeformed samples, as illustrated in Figure 1. There are coarse plate-like eutectic Si particles which maybe cause the stress concentration in the α(Al) matrix under the action of stress, as shown in Figure 1a,b. The morphology of eutectic Si particles of Sr-modified-undeformed is spherical or elliptical, as shown in Figure 1c,d.

**Figure 2:**
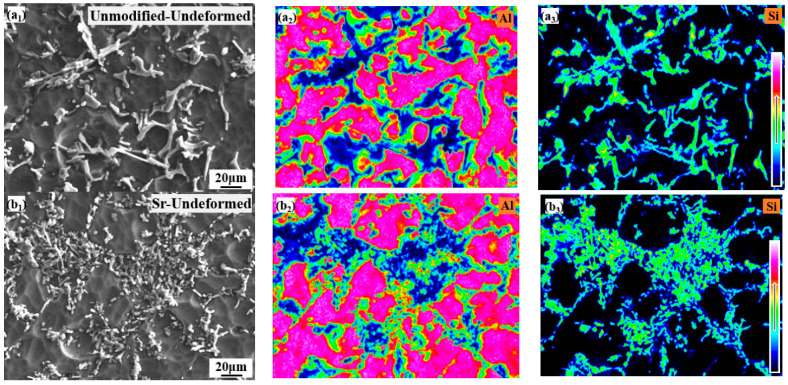
Distribution of the undeformed samples in the initial microstructures: (**a_1_**–**a_3_**) unmodified-undeformed; (**b_1_**–**b_3_**) Sr-modified-undeformed.

The distribution and enrichment of Al and Si elements of the undeformed samples in the initial microstructures is shown in Figure 2. The enrichment intensity of Al and Si elements in the alloy is similar, but the distribution of eutectic Si particles is biased.

**Figure 3:**
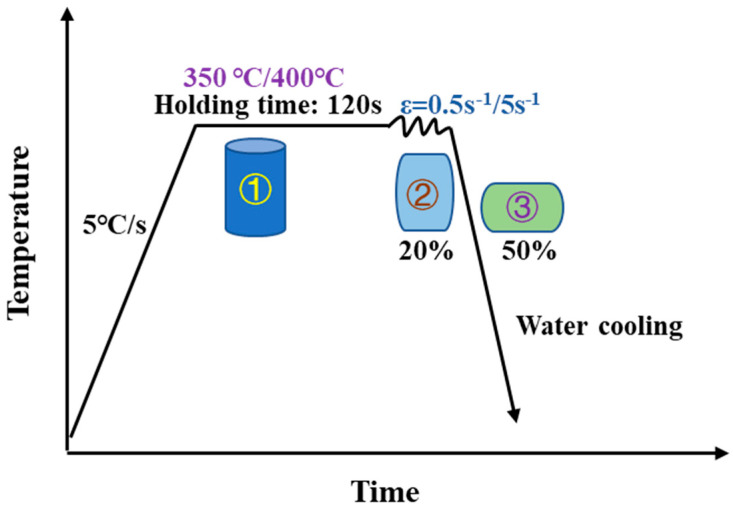
Schematic diagram of thermal compression process.

**Figure 4:**
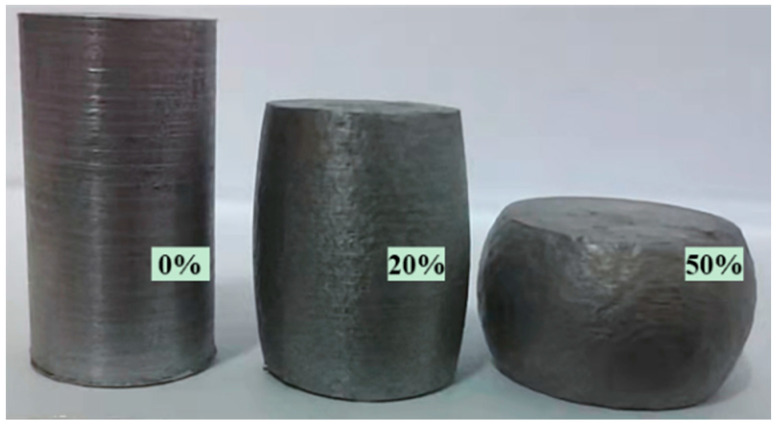
Samples before and after thermal compression process.

That is a schematic diagram of thermal compression process of the tested Al-Si alloys is shown in Figure 3. The hot-compressed samples with a height of 15 mm and a diameter of 8 mm were processed by wire cutting. The hot compression tests were performed by simulation equipment. For obtain a microstructure with nearly the same internal temperature of the samples before deformation, the samples were heated to a set temperature at a rate of 5 °C/s, and the samples were held for 120 s. Then, the samples were deformed at a selected strain rate and temperature. Deformed samples were quenched with cold water immediately to study the deformed microstructures, and. the samples were cut along the axial direction. The deformation temperatures of hot compression test were 350 °C and 400 °C, the deformations were 20% and 50%, and the strain rates were 0.5 s^−1^ and 5 s^−1^. Figure 4 is the picture of the samples before and after compression deformation.

**Figure 5:**
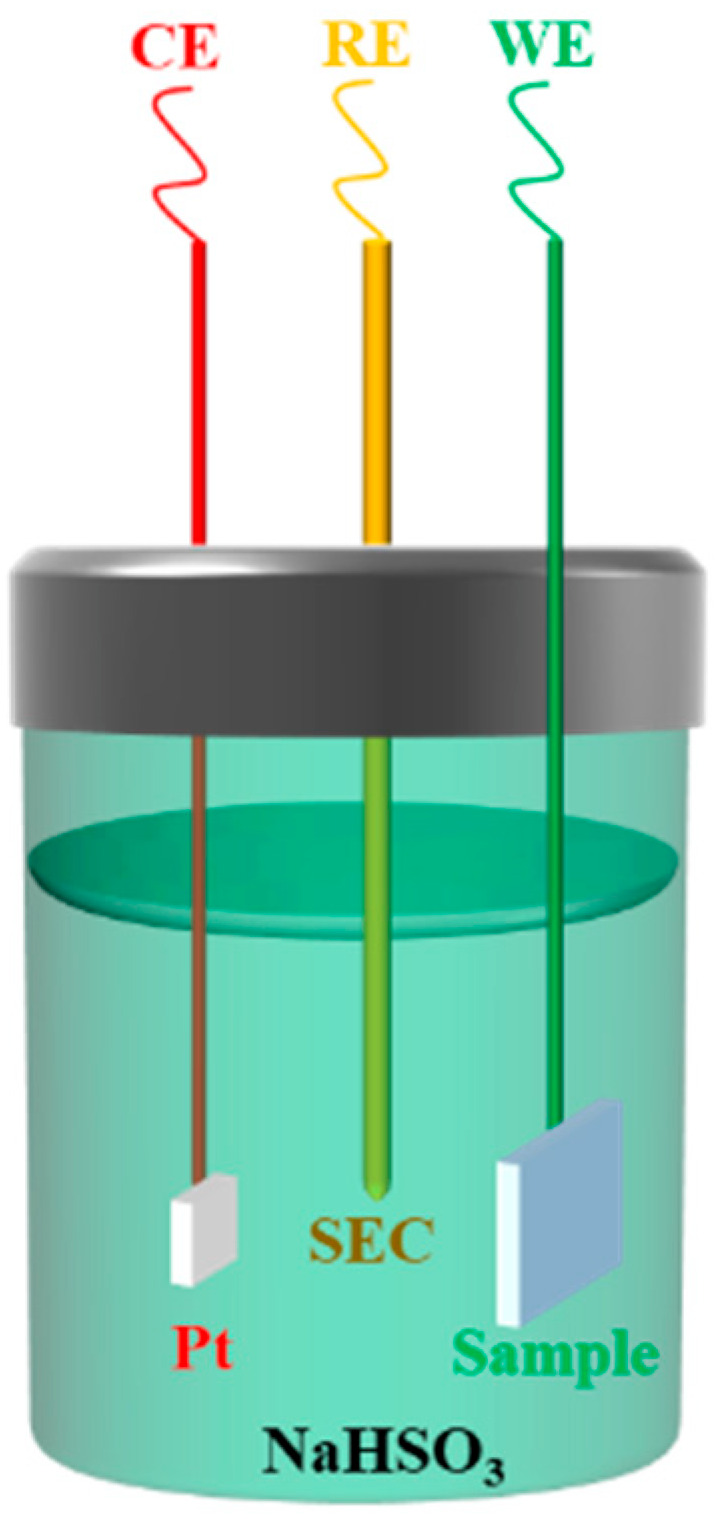
Schematic diagram of the three-electrode system.

The CS2350 dual-cell electrochemical workstation (Wuhan Corrtest Instruments Corp., Ltd., Wuhan, China) was used to measure the polarization curve of the samples. The electrode system was a three-electrode system, and the electrolyte was 0.01 mol/L NaHSO_3_ solution, as shown in Figure 5. The samples were WE (working electrode). The saturated calomel electrode (SEC) was RE (reference electrode), and the CE (counter electrode) was Pt in the three-electrode system. The scanning speed was 0.5 mv/s.

## 3. Results and Discussion

### 3.1. Flow Stress Behavior

**Figure 6:**
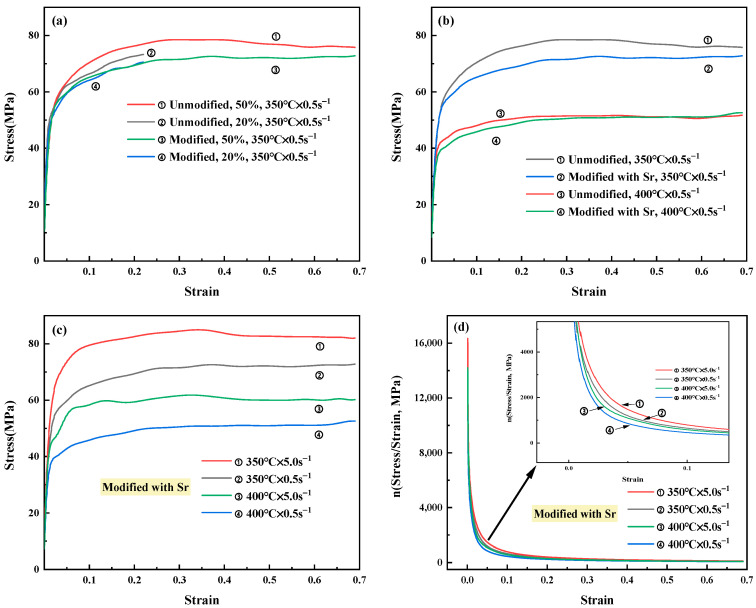
The relationship among the stress, temperatures, and strain rates of the tested Al-7Si alloy: (**a**) flow stress-strain curves of the tested alloys at different strains at 350 °C × 0.5 s^−1^; (**b**) flow stress-strain curves of the tested alloys at different temperatures at 0.5 s^−1^; (**c**) flow stress-strain curves of Sr-modified-Al-7Si alloy at different temperatures and strain rates; (**d**) strain hardening exponent “*n*” of Sr-modified-Al-7Si alloy at different conditions.

The curves of true stress-true at the different strain rates, temperatures, and strains of the tested alloy are shown in Figure 6. There was a typical characteristic of yielding and strain hardening in the curves. The stress rose rapidly with the increase of strain, and after reaching the peak stress. Then, the stress basically did not change obviously with the further increase of strains in Figure 6.

The stress-strain curve of Sr-modified-Al-7Si alloy at the condition of 20% deformation basically coincided with that at the condition of 50% deformation at the same temperature and strain rate in Figure 6a. However, the coincidence degree in different deformations of stress strain curves of unmodified-Al-7Si alloy was lower than that of Sr-modified-Al-7Si alloy at the same strain rate and temperature. Furthermore, the peak stress of unmodified-Al-7Si alloy was higher than that of Sr-modified-Al-7Si alloy at the same deformation condition in Figure 6b. Some studies [37] have pointed out that the stress required for particles cracking decreases with the increasing of particle size, the number of cracked-particles increases with the strain and stress increases, and the longish particles are more prone to cracking than spherical particles. As shown in Figure 1 and Figure 2, almost all Si particles of Sr-modified-Al-7Si alloy were fine and evenly distributed. The stress of eutectic Si particles in Sr-modified-Al-7Si alloy was lower than that of the unmodified Al-7Si alloy, and the cracking probability of Si particles was also lower. The stress concentration in the α(Al) matrix of Sr-modified-Al-7Si alloy was significantly less than that of unmodified Al Si alloy in the plastic deformation process, and it is accord with the results obtained in Figure 6a,b.

The peak stress of Sr-modified-Al-7Si alloy increased with the increase of the decrease of deformation temperatures and strain rates in Figure 6c. This indicates that the stress-strain curves of Al-7Si alloy are very sensitive to temperatures, strain rates, and microstructures. Figure 6d shows the strain hardening values *n* of Sr-modified-Al-7Si alloy at the different conditions. With the increase of strains, the value of *n* decreased first and then tended to be stable. The value of *n* was affected by temperatures and strain rates, especially in the range of 0.02–0.08 strain of Sr-modified-Al-7Si alloy in Figure 6d. In other words, decreasing temperature and increasing strain rate made the *n* value higher in the range of 0.02–0.08 strain. The existence of these phenomena is supposed to be relevant to the morphology and uneven distribution of Si particles. Hence, it is essential to analyze further the microstructures after deformation.

### 3.2. Microstructures Evolution

**Figure 7:**
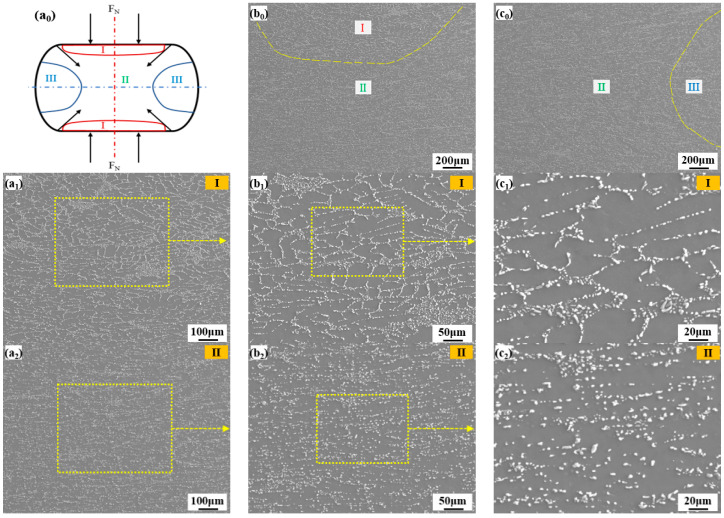

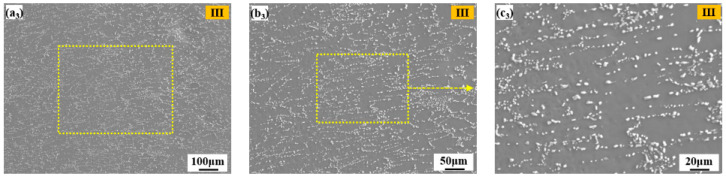
Microstructures of Sr-modified alloy at 400 °C × 0.5 s^−1^: (**a_0_**) schematic diagram of the deformed samples; (**b_0_**,**c_0_**) microstructures at different deformation zones; (**a_1_**,**b_1_**,**c_1_**) hard deformation zone I; (**a_2_**,**b_2_**,**c_2_**) homogeneous deformation zone II; (**a_3_**,**b_3_**,**c_3_**) free deformation zone III.

There is friction between the compression hammer and the cylindrical samples at both ends of the thermal simulation testing machine, and there is gradient of temperature between the middle and the end face of the samples, so that the stress distribution in the samples is not uniform, resulting in uneven deformation. Therefore, the microstructures of the samples can be divided into three regions [38]. The deformation partition of the compressed samples at 400 °C and 0.5 s^−1^ is taken as an example, as shown in Figure 7.

Generally, the microstructures of zone II that can be deformed uniformly are used to analyze the law of specific state. It can be found that the α(Al) grains are elongated after deformation. The Si particles distribution in the homogeneous deformation zone II is more even than that in zone I and zone III. The deformation of A356 at high temperature is mainly concentrated in the soft phase α(Al) matrix, which has a high lamination energy and is prone to cross slip of dislocation. Therefore, when the strain reaches a certain degree, the strain hardening and the softening of dynamic recovery reach a balance, and a stable flow stress platform appears on the curve, which is accord with the results obtained in Figure 6. The strain hardening of the matrix is estimated to be affected by interaction of dislocation to dislocation, dislocation density and hinder of the plastic flow owing to the drag force provided by Si particles [27,39].

**Figure 8:**
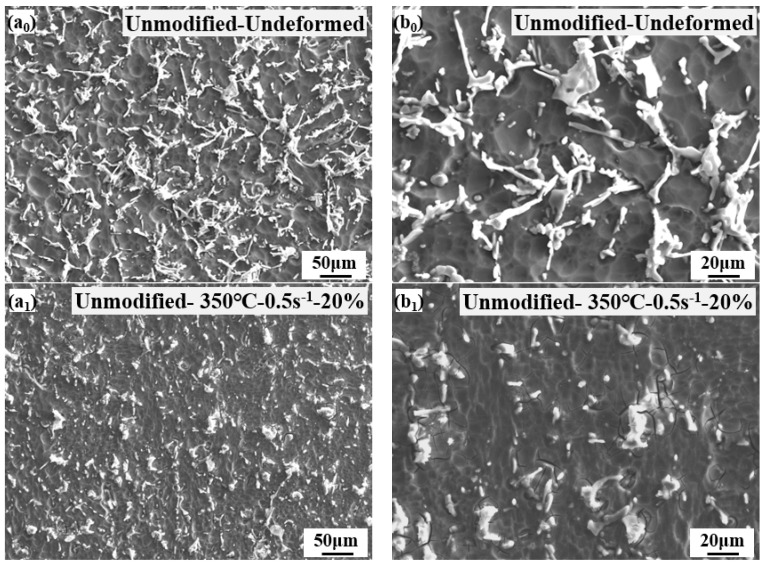

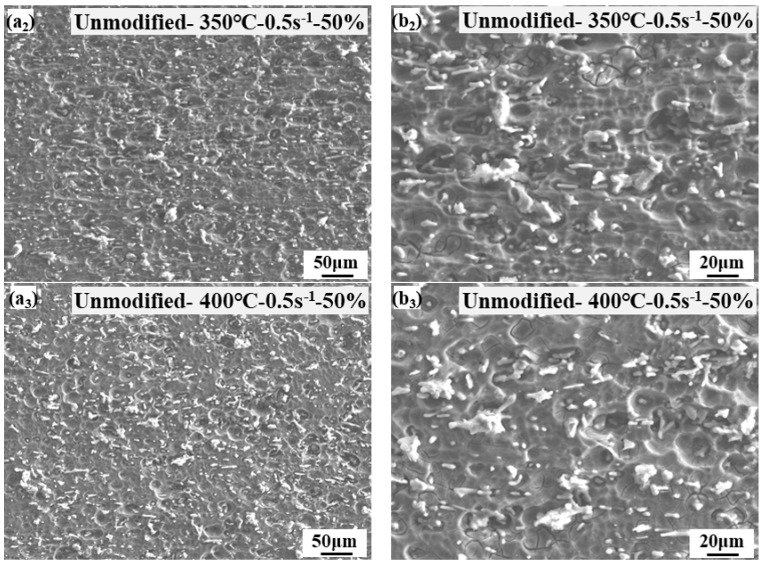
Microstructures of unmodified-Al-7Si alloy in homogeneous deformation zone II at different deformation conditions: (**a_0_**,**b_0_**) unmodified-undeformed; (**a_1_**,**b_1_**) unmodified-350 °C-0.5 s^−1^-20%; (**a_2_**,**b_2_**) unmodified-350 °C-0.5s^−1^-50%; (**a_3_**,**b_3_**) unmodified-400 °C-0.5 s^−1^-50%.

The morphology of unmodified-Al-7Si alloy in homogeneous deformation zone II at different deformation conditions were analyzed and compared in Figure 8. Compared with the undeformed-unmodified-Al-7Si alloy in Figure 8a_0_,b_0_, the thermal deformation caused the Si particles to be broken and distributed more evenly in Figure 8a_1_–a_3_,b_1_–b_3_. Compared with the lower deformation of 20%, the higher deformation of 50% was more beneficial to make distribution of the eutectic Si particles more even in Figure 8a_1_,a_2_,b_1_,b_2_. In fact, the strain was passed through the α(Al) matrix to the brittle Si particles during plastic deformation. The Si particles cannot adjust the plastic deformation of the α(Al) matrix, therefore an inhomogeneous strain accumulation occurred in both of the α(Al) matrix and the Si particles. This leads to stress increasing rapidly at low strains and may give rise to cracking of the Si particles [27].

**Figure 9:**
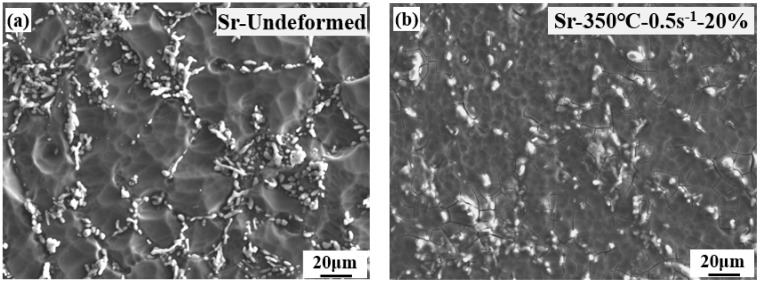

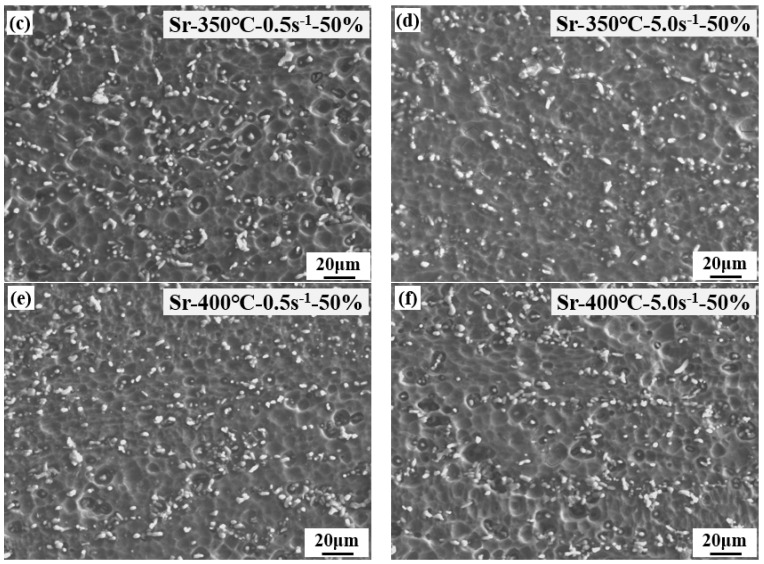
Microstructures of Sr-modified-Al-7Si alloy in homogeneous deformation zone II at different deformation conditions: (**a**) Sr-undeformed; (**b**) Sr-350 °C-0.5 s^−1^-20%; (**c**) Sr-350 °C-0.5 s^−1^-50%; (**d**) Sr-350 °C-5.0 s^−1^-50%; (**e**) Sr-400 °C-0.5 s^−1^-50%; (**f**) Sr-400 °C-5.0 s^−1^-50%.

The morphology of Sr-modified-Al-7Si alloy in homogeneous deformation zone II at different conditions are analyzed and compared in Figure 9. Although the morphology of eutectic Si particles in the microstructures of Sr-undeformed alloy was refined, there was still some segregation phenomenon of eutectic Si particles in Figure 9a. The deformation made the distribution of Si particles more even in Sr-modified-Al-7Si alloy in Figure 9b–f. The increase of deformation was beneficial to the even distribution of Si particles at the same strain rate and temperature in Figure 9a,b. There was a slight decrease in the size of Si particles. The strain rates had a limited effect on the distribution and morphology of Si particles of Sr-modified-Al-7Si alloy in Figure 9.

### 3.3. Corrosive Properties

**Figure 10:**
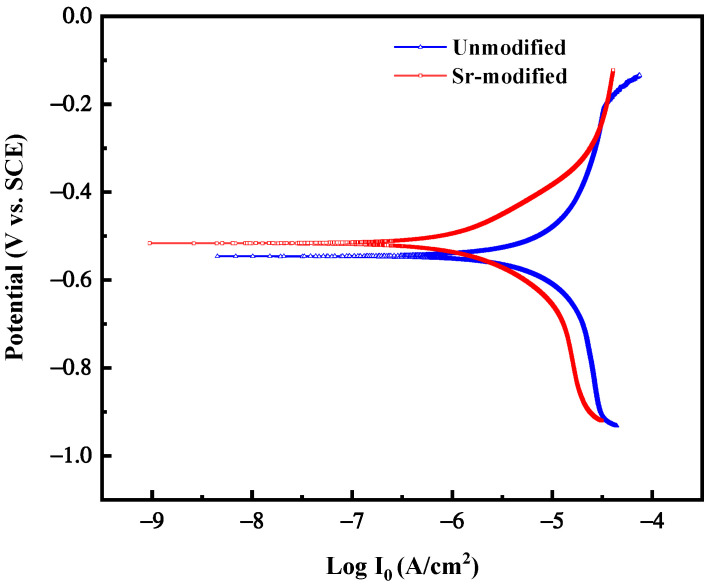
Polarization curves of Al-7Si alloy samples tested in 0.01 mol/L NaHSO_3_ solution at 25 °C.

The polarization curves measurement tests were implemented to study the effect of Si particles on the corrosion properties. The polarization curves of unmodified-Al-7Si alloy and Sr-modified-Al-7Si alloy are shown in Figure 10. The polarization curve is the characterization of the corrosion relation curve of electrode potential and current density [40]. Parameter values obtained from polarization curves are shown in Table 2. It should be noted that the morphology and distribution of Si particles had a certain impact on the corrosive properties of unmodified-Al-7Si alloy and Sr-modified-Al-7Si alloy. The self-corrosion current I_corr_ of Sr-modified-Al-7Si alloy was lower than that of the unmodified-Al-7Si alloy, and the self-corrosion potential E_corr_ of Sr-modified-Al-7Si alloy was higher than that of unmodified-Al-7Si alloy.

The measurement of corrosion potential of aluminum alloy is of great meaning to the study of its corrosive properties, which reflects the tendency of the alloy to dissolve in solution. In view of the results in Figure 10 and Table 2, it can be found that the corrosive properties of the Sr-modified-Al-7Si alloy was better than that of unmodified-Al-7Si alloy. As we all know, the Si particles as the cathode, and Si particles and α(Al) matrix form an electrode galvanic pair, which easily produces pitting corrosion [29]. The corrosion starts from Si particles and grain boundaries preferentially and is mainly pitted. In solid state, the concentration of Si element in α(Al) matrix is very low, and it is prone to precipitate at the grain boundary in needle-like or slate-like morphology in the unmodified-Al-7Si alloy. At the same time, the coarse phase interface between the coarse Si particles and α(Al) matrix has an adverse impact on the corrosive properties of the alloy, which destroys the continuity of the aluminum protective film and makes the corrosion easier. Compared with unmodified-Al-7Si alloy, the size of Si particles is smaller and the distribution is more even in Sr-modified-Al-7Si alloy, which is beneficial to improve the corrosive properties of Al-Si alloy.

## 4. Conclusions

(1) The peak stress of unmodified-Al-7Si alloy is obviously higher than that of Sr-modified-Al-7Si alloy at the same deformation conditions, and this is related to the morphology and distribution of Si particles. The stress-strain curves of Al-7Si alloy are very sensitive to temperatures, strain rates and microstructures. With the increase of strains, the values of strain hardening *n* decreases first and then tends to be stable.
(2) The morphology of Si particles of unmodified-Al-7Si alloy changes from coarse plate-like to short-rod after deformation, and the distribution of Si particles in homogeneous deformation zone II becomes more even. The increase of deformation is beneficial to the distribution of Si particles more even at the same temperature and strain rate. The strain rates have little effect on the morphology and distribution of Si particles.
(3) The electrochemical self-corrosion potential of Sr-modified-Al-7Si alloy is higher than that of the unmodified-Al-7Si alloy. It indicates that the distribution and morphology of Si particles have a certain influence on the corrosion properties of Al-Si alloy. That is, the even fine Si particles morphology is more conducive to improving the corrosive properties of the tested Al-7Si alloy.

## Figures and Tables

**Table 1:** The chemical composition of the tested alloys (wt.%).

Element	Si	Mg	Ti	Fe	Sr	Al
unmodified-Al-7Si alloy	7.22	0.32	0.20	0.111	-	Bal.
Sr-modified-Al-7Si alloy	7.24	0.31	0.19	0.110	0.3	Bal.

**Table 2:** Parameter values obtained from polarization curves of unmodified-Al-7Si alloy and Sr-modified-Al-7Si alloy in 0.01 mol/L NaHSO_3_ solution at 25 °C.

Samples	I_corr_ (μA/cm^2^)	E_corr_ (V vs. SCE)	Corrosion Rate (mm/a)
Unmodified	4.4537	-0.54101	0.05205
Sr-modified	1.7012	-0.51621	0.01988

## Data Availability

All data are presented within the manuscript.
